# A novel anti-*Toxoplasma* peptide suppresses parasite invasion and rescues host autophagic defenses

**DOI:** 10.1128/spectrum.02218-25

**Published:** 2025-12-29

**Authors:** Ji Zhao, Baocan Zhuang, Yun Yang, Elvis Quansah, Yuanyuan Cao, Qingli Luo, Jilong Shen, Li Yu

**Affiliations:** 1 Department of Microbiology and Parasitology, First Affiliated Hospital of Anhui Medical University 12485, Hefei, China; 2 Department of Laboratory Medicine, First Affiliated Hospital of Anhui Medical University 12485, Hefei, China; 3 Anhui Provincial Key Laboratory of Zoonoses, School of Basic Medical Sciences, Anhui Medical University 12485, Hefei, China; 4 Department of Biological Science Education, Akenten Appiah-Menka University of Skills Training and Entrepreneurial Development, Mampong-Asante, Ghana; Clemson University, Clemson, South Carolina, USA

**Keywords:** autophagy, TgMIC6, peptide, *Toxoplasma gondii*

## Abstract

*Toxoplasma gondii,* a ubiquitous intracellular protozoan parasite, poses life-threatening risks to immunocompromised hosts. Current first-line treatments for toxoplasmosis are limited by significant toxicity and high post-treatment recurrence rates. In this study, we employed phage display technology to identify peptides targeting TgMIC6 and disrupting its immune evasion function. Among these, the C8 peptide exhibited dual efficacy: it not only inhibited *T. gondii* invasion *in vitro* but also restored host autophagy, countering the parasite’s immune escape mechanisms. Furthermore, *in vivo* studies confirmed the potent parasiticidal activity of C8 against multiple *T. gondii* strains. These findings highlight C8 as a promising therapeutic candidate for toxoplasmosis.

**IMPORTANCE:**

The study identifies the C8 peptide as a novel anti-*Toxoplasma* agent with dual efficacy: it directly inhibits parasite invasion and restores host autophagy compromised by *T. gondii* immune evasion. Demonstrating potent parasiticidal activity *in vitro* and *in vivo*, C8 significantly prolongs survival in acute infection models across multiple strains without cytotoxicity. Its multi-target mechanism and favorable safety profile address critical limitations of current therapies. While stability and chronic infection efficacy require optimization, C8 represents a promising peptide-based therapeutic candidate, offering a foundation for developing next-generation anti-toxoplasmosis drugs with enhanced specificity and reduced side effects. This work highlights the potential of peptide biologics in combating apicomplexan infections.

## INTRODUCTION

*Toxoplasma gondii* is an obligate intracellular parasitic eukaryotic protozoan that can infect almost all warm-blooded animals (1). *T. gondii* infection usually presents as a latent infection without obvious clinical symptoms in immunocompetent individuals but can form cysts in the brain and muscles that persist within the host for life (2). However, in immunocompromised individuals, such as those with AIDS or organ transplantation, the cysts easily morph into tachyzoites and cause acute infections with severe clinical manifestations (3). *T. gondii* is teratogenic and can be transmitted vertically from a pregnant woman to the fetus, resulting in miscarriage or congenital malformations (4–6). Early genotyping studies of *T. gondii* were conducted on three classical lineages isolated from North America and Europe, including type I, type II, and type III strains; the first two are usually isolated in humans (7). Chinese1 (ToxoDB#9) is the most prevalent lineage in China, and it comprises the highly virulent strain, TgCtwh3 (WH3), and the low virulent strain, TgCtwh6 (WH6) (8–10).

Current chemotherapeutic options for toxoplasmosis are limited. The frontline treatment for toxoplasmosis is a combination of pyrimethamine and sulfadiazine, which synergistically target folate metabolism. However, this regimen is associated with adverse effects, including hematologic toxicity, hypersensitivity, and teratogenicity, limiting its use in pregnant women (11–14). Alternative therapies, such as pyrimethamine combined with clindamycin, azithromycin, or atovaquone (15–18), offer partial solutions but fail to eliminate tissue cyst (19–21), necessitating long-term maintenance therapy in immunocompromised patients. Recent advances in toxoplasmosis chemotherapy have focused on developing novel compounds and optimizing existing treatments, particularly targeting the mitochondrial electron transport chain (22–24), endochin-like quinolones (ELQs) including the bioavailable prodrug (25), calcium-dependent protein kinase inhibitors (26–28), and next-generation antifolates (29) that outperform pyrimethamine in selectivity and potency against both tachyzoite and bradyzoite forms. Despite these advancements, several challenges remain in the development of effective chemotherapeutic agents for toxoplasmosis. One notable issue is the emergence of resistance to these compounds (24, 30). These challenges highlight the urgent need to explore alternative therapies.

Peptide-based therapeutics represent a unique class of pharmaceutical agents that have gained widespread clinical application in oncology, virology, and bacteriology due to their exceptional target specificity, high binding affinity, and favorable toxicity profiles (31–34). Emerging evidence demonstrates their therapeutic potential against *T. gondii* infection. For instance, cal14.1a, a bioactive peptide derived from *Conus californicus*, significantly inhibits *T. gondii* host cell invasion and intracellular proliferation (35). Similarly, longicin P4, an antimicrobial peptide isolated from *Haemaphysalis longicornis*, exhibits potent growth-suppressive activity against *T. gondii* (36). Furthermore, the α-helical cationic peptides HPRP-A1 and HPRP-A2, originally identified in *Helicobacter pylori*, effectively impair tachyzoite viability and block their adhesion to and invasion of macrophages (37). Notably, the spider venom-derived peptide XYP1 and its optimized derivatives (XYP1-18 and XYP1-18-1) from *Lycosa coelestis* demonstrate robust anti-*Toxoplasma* activity through immunomodulation of host inflammatory responses (38, 39). Collectively, these findings underscore the considerable therapeutic promise of peptide-based strategies for combating *T. gondii* infection.

Microneme protein 6 of *T. gondii* (TgMIC6) is a transmembrane protein that forms a complex with MIC4 and MIC1, playing a critical role in host cell invasion (40–43). Additionally, TgMIC6 contains multiple epidermal growth factor (EGF)-like domains, which bind to host epidermal growth factor receptor (EGFR) and suppress cellular autophagy, thereby enabling the parasite to evade host immune clearance (44). Given its essential role in *Toxoplasma* infection, we previously identified a panel of high-affinity peptides targeting TgMIC6 using phage display library screening. In this study, we further evaluated their anti-*Toxoplasma* efficacy and demonstrated that the C8 peptide conferred robust protection in mice against challenge with multiple *T. gondii* strains, highlighting its therapeutic potential.

## RESULTS

### Screened peptides inhibit *T. gondii* invasion *in vitro*

**Fig 1:**
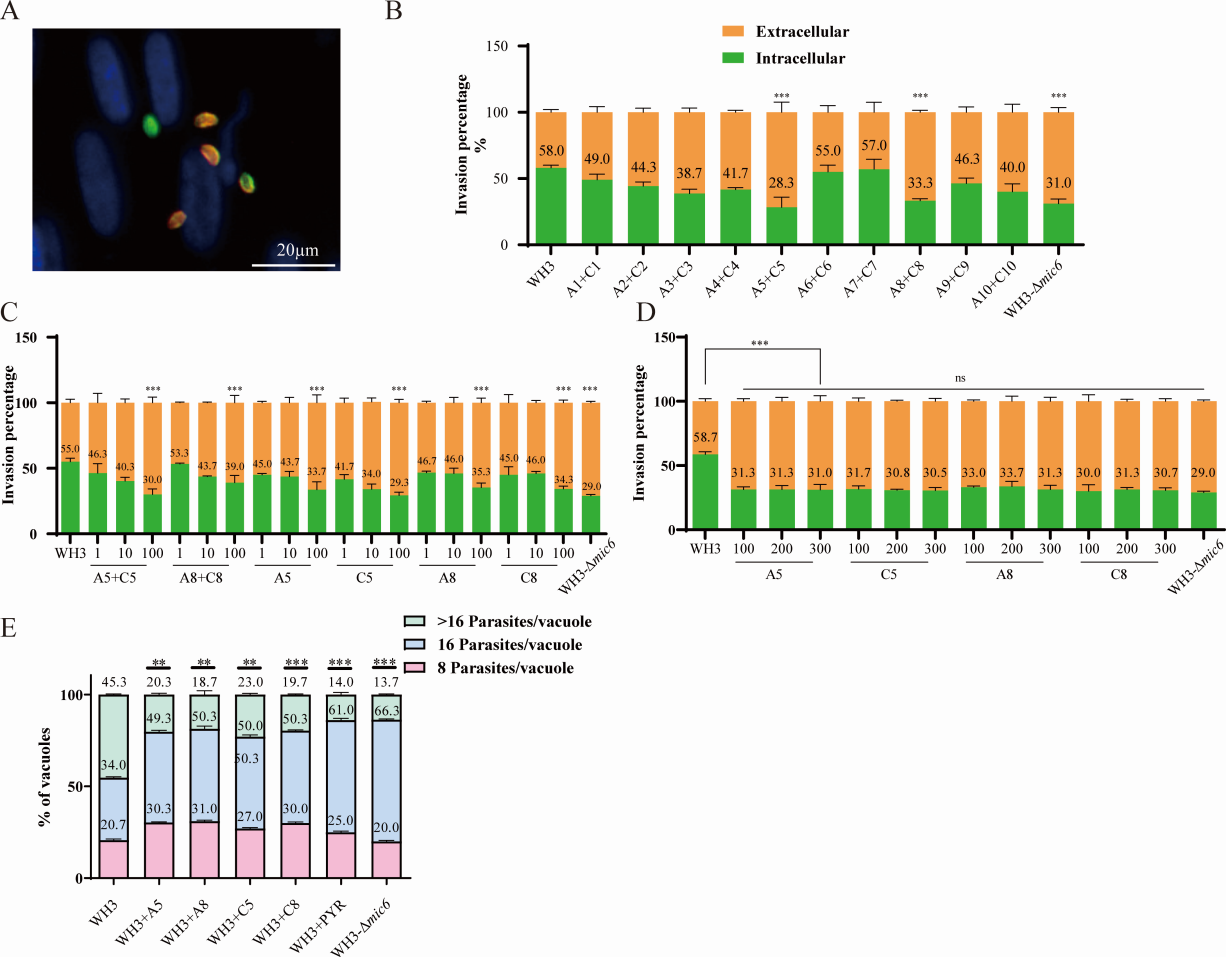
Anti-*Toxoplasma* activity of selected peptides *in vitro*. (**A**) Immunofluorescence analysis of *T. gondii* invasion in HFF monolayers. Intracellular tachyzoites (green) and extracellular parasites (yellow) were distinguished by differential antibody staining post-permeabilization. Scale bar: 20 μm. (**B–D**) Quantitative assessment of invasion inhibition. (**B**) Percentage of invaded tachyzoites following treatment with 100 μg/mL peptide candidates (A1-C10) relative to untreated WH3 strain controls. (**C and D**) Dose-dependent inhibition by lead peptides (A5, C5, A8, C8) across concentration gradients (1, 10, 100 μg/mL). All invasion assays (**B–D**) show mean ± SD of triplicate experiments analyzed by two-tailed unpaired *t*-test (***P < 0.001 vs WH3 control), ns, not significant (*P* > 0.05). (**E**) Intracellular replication analysis after 36 h infection. Histograms depict the percentage of parasitophorous vacuoles containing ≥ 16 tachyzoites in peptide-treated (100 μg/mL) vs control groups (**P < 0.01, ***P < 0.001 vs WH3 control). Data represent mean ± SEM from ≥ 100 vacuoles per condition.

To identify peptides capable of inhibiting *T. gondii* host cell invasion *in vitro*, we systematically screened 20 candidate peptides (Fig. S1) administered in pairwise combinations at standardized concentrations of 100 μg/mL. Through this combinatorial screening approach, two peptide pairs (A5+C5 and A8+C8) demonstrated significant anti-invasion activity against *T. gondii* tachyzoites (Fig. 1B). Subsequent dose-response analyses were performed using three concentration gradients (1, 10, and 100 μg/mL) of individual peptides (A5, C5, A8, C8) administered either singly or in combination. Quantitative assessment revealed concentration-dependent inhibition of parasite invasion for all four peptides, with maximal efficacy achieved at 100 μg/mL (Fig. 1C and D). Importantly, peptide treatments at these concentrations showed no detectable cytotoxicity to host cells as determined by viability assays (Fig. S2A). To further characterize the anti-parasitic effects, we evaluated intracellular replication dynamics using WH3 strain tachyzoites, demonstrating that peptide treatment significantly reduced parasite proliferation within PVs, with particularly robust inhibition observed for C5 and C8 peptides (Fig. 1E).

### Peptide treatment controls acute toxoplasmosis in mice

**Fig 2:**
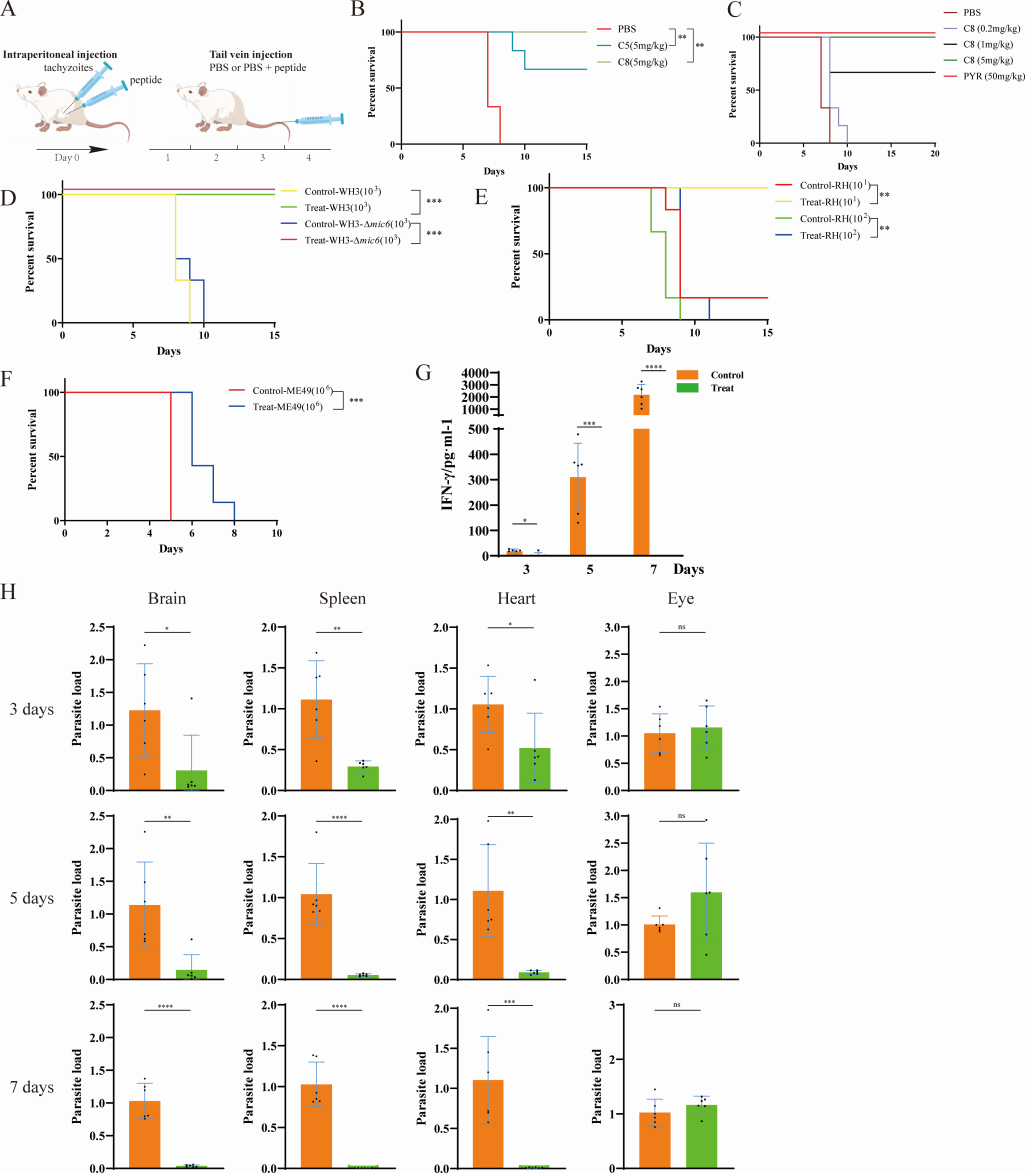
Therapeutic efficacy of C8 peptide in murine models of acute toxoplasmosis. (**A**) Experimental design schematic: BALB/c mice were intraperitoneally infected with *T. gondii* tachyzoites (day 0) and treated with either C5 or C8 peptide (5 mg/kg) via initial intraperitoneal injection followed by daily intravenous administration for 4 consecutive days. Control groups received equivalent volumes of sterile PBS. (**B–F**) Kaplan-Meier survival analysis. (**B**) Comparative survival of WH3 strain-infected mice (1 × 10^3^ tachyzoites) treated with C5 or C8 peptide (5 mg/kg) versus PBS control (*n* = 6/group). (**C**) Dose-response of C8 peptide (0.2, 1, 5, 10 mg/kg) against WH3 infection (1 × 10^3^ tachyzoites; *n* = 6/group). (**D–F**) Strain-specific efficacy against varying infectious doses, *n* = 6/strain. (**D**) WH3 and WH3-Δ*mic6*, (**E**) RH, (**F**) ME49. Statistical significance was determined by Gehan-Breslow-Wilcoxon test (**P < 0.01, ***P < 0.001 vs PBS controls). (**G**) Serum IFN-γ levels measured by ELISA at days 3, 5, and 7 post-WH3 infection (1 × 10^3^ tachyzoites) in peptide-treated versus control mice. Data represent mean ± SEM (*n* = 6/timepoint; *P < 0.05, ***P < 0.001, ****P < 0.0001 by two-way *ANOVA*). (**H**) Tissue parasite burden quantification: qPCR analysis of *T. gondii* B1 gene copy number normalized to 100 ng total DNA in brain, spleen, heart, and eye at day 3, 5, and 7 post-infection. ns, not significant (*P* > 0.05). Bars represent mean ± SEM.

To evaluate the therapeutic potential of candidate peptides against acute *T. gondii* infection, BALB/c mice were intraperitoneally inoculated with 1 × 10^3^ WH3 strain tachyzoites and subsequently administered C8 peptide via an initial intraperitoneal bolus (5 mg/kg) followed by daily tail vein injections of the same dose for five consecutive days (Fig. 2A). Comparative efficacy analysis revealed superior therapeutic outcomes with C8 peptide compared to C5 at equivalent concentrations (5 mg/kg), as evidenced by significantly prolonged survival rates (Fig. 2B). Dose-response studies established 5 mg/kg as the optimal therapeutic concentration for complete resolution of acute infection (Fig. 2C), with no observed *in vivo* cytotoxicity at this dosage (Fig. S2B).

To assess strain-specific efficacy, we challenged BALB/c mice with four genetically distinct *T. gondii* strains at varying infectious doses: WH3 (1 × 10^3^ tachyzoites), WH3-Δ*mic6* (1 × 10^3^ tachyzoites), RH (1 × 10^1^ and 1 × 10^2^ tachyzoites), and ME49 (1 × 10^6^ tachyzoites). C8 peptide treatment consistently extended survival across all strains, achieving complete cure in WH3-Δ*mic6*-infected mice (Fig. 2D through F). Longitudinal analysis demonstrated that C8 treatment maintained serum IFN-γ levels and significantly reduced parasite burdens in the brain, spleen, and heart compared to PBS-treated controls at days 3, 5, and 7 post-infection (Fig. 2G and H). In contrast, a non-significant trend toward increased parasite burden was observed in the eyes of C8-treated mice. However, in a parallel chronic infection model orally inoculated with 20 WH6 tissue cysts, C8 peptide failed to reduce cyst burden or prevent chronic infection establishment (Fig. S3A and B).

### C8 peptide inhibits *T. gondii* invasion by binding to invasion-related proteins of the parasite

**Fig 3:**
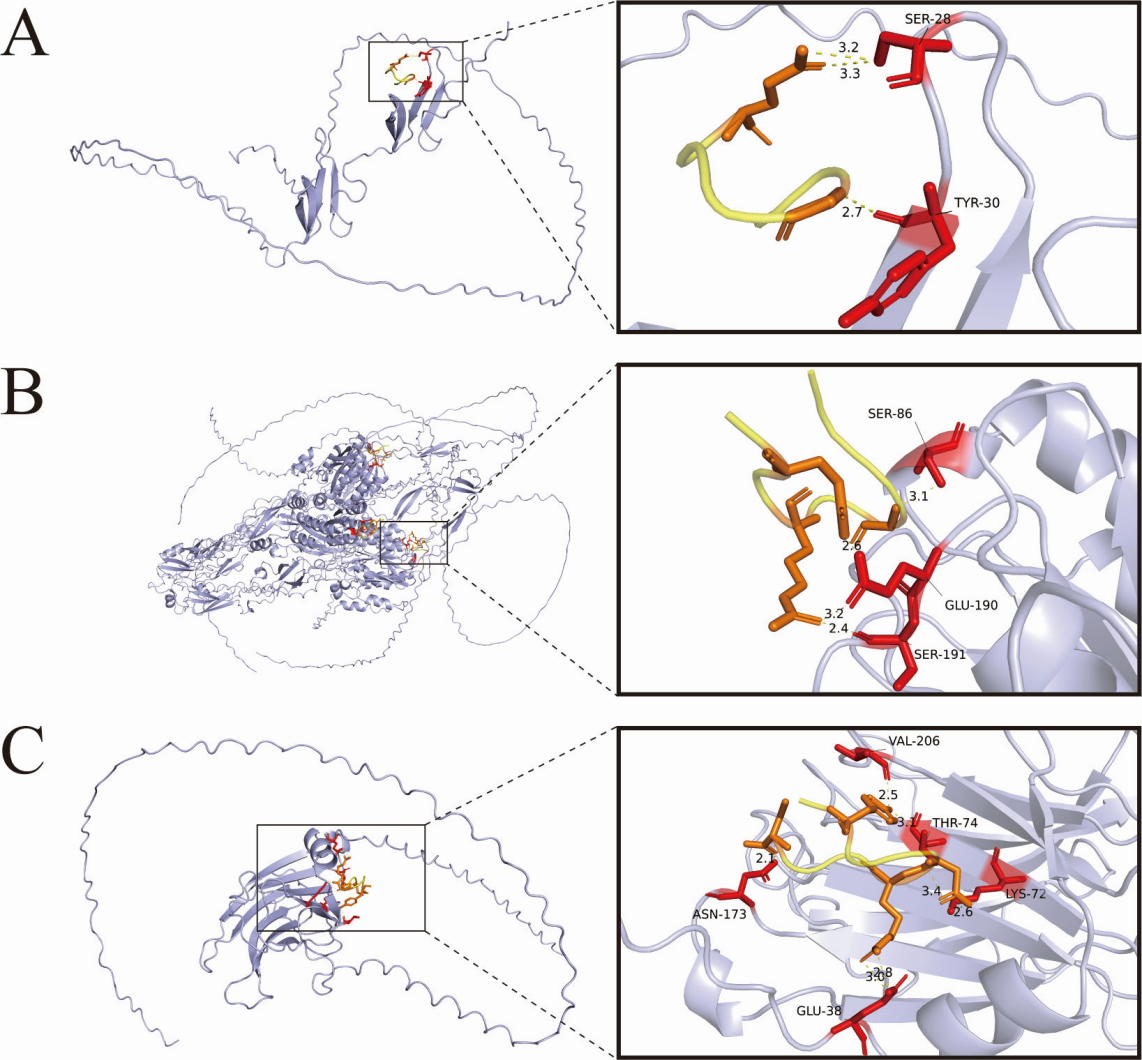
Structural characterization of C8 peptide interactions with invasion-associated proteins. (**A**) Molecular docking model of C8 peptide bound to the EGF-like domain of TgMIC6. (**B**) Predicted binding interface between C8 peptide and the vWA domain of TgMIC2. (**C**) Simulation of C8 peptide binding to the M2AP apical complex protein. All models were generated using AlphaFold3 with molecular dynamics refinement. Distance measurements (Å) indicate lengths between interacting heavy atoms. Color scheme: C8 peptide (yellow), interacting peptide residues (orange), protein binding site residues (red).

In the murine infection model, therapeutic administration of the C8 peptide demonstrated equivalent efficacy in WH3-Δ*mic6* strain-infected mice compared to wild-type WH3 strain-infected animals, suggesting potential alternative mechanisms of action beyond TgMIC6 interaction. Computational simulations predicted that the C8 peptide exhibits binding promiscuity, potentially interacting with both parasite-derived invasion machinery and host cell surface molecules. Molecular docking analysis identified a potential specific interaction between peptide C8 and the epidermal growth factor (EGF)-like domain of TgMIC6 (Fig. 3A). Additionally, high-affinity binding sites were observed on key invasion-related proteins, including TgMIC2 and its partner protein TgM2AP (Fig. 3B and C). Structural modeling further revealed that the C8 peptide forms stable interactions with the von Willebrand factor A (vWA) integrin-like A domain (A/I) of TgMIC2 (45–47), a critical functional domain mediating host cell adhesion during invasion. These multi-target binding characteristics likely contribute to the peptide’s potent invasion-inhibitory activity through simultaneous disruption of multiple molecular interactions essential for parasite entry.

### C8 peptides rescue host autophagic defense

**Fig 4:**
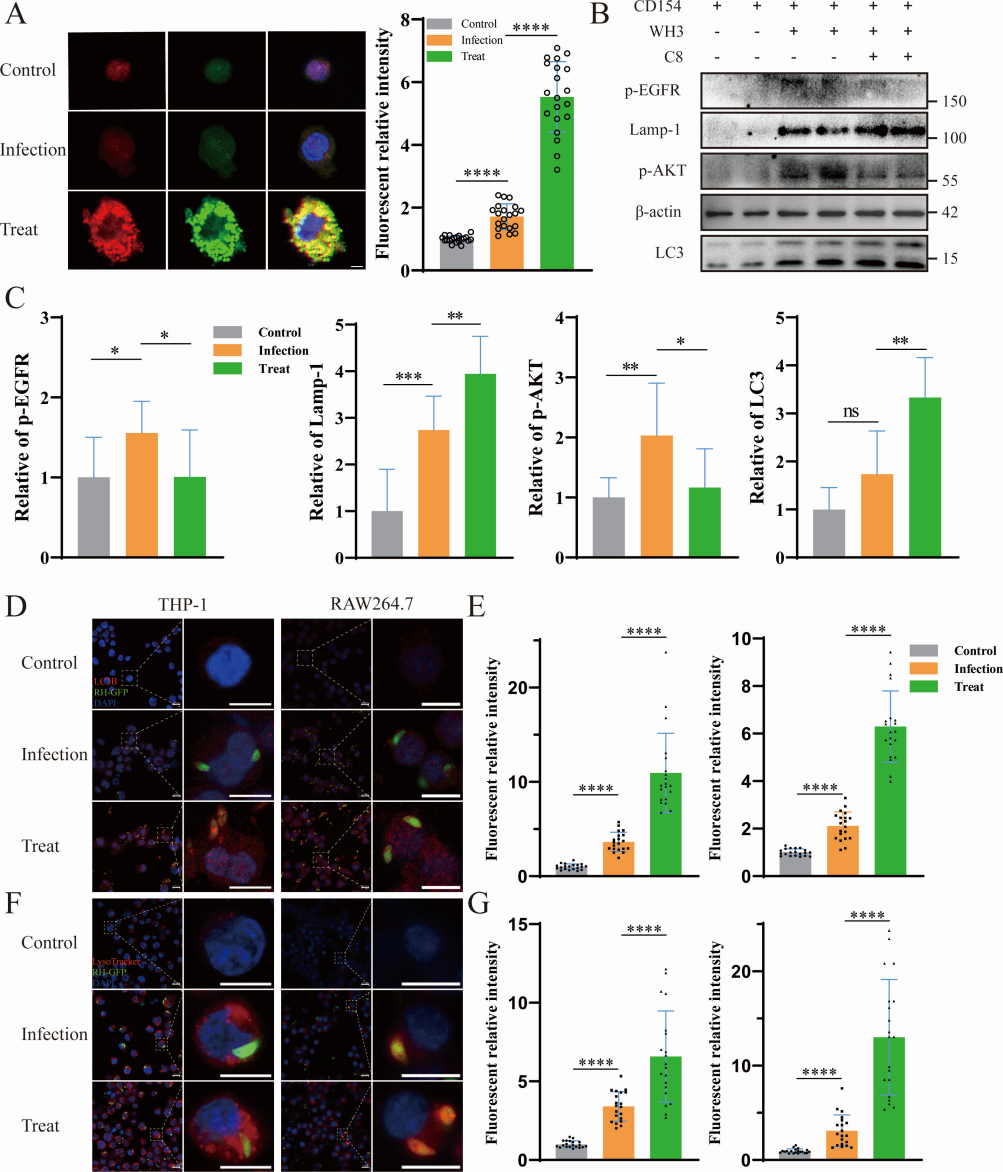
C8 peptide restores anti-*Toxoplasma* autophagy pathways compromised by parasite immune evasion mechanisms. (**A**) Autophagic flux analysis in PMA-differentiated THP-1 transduced with adenoviral RFP-GFP-LC3 reporter and human CD154 stimulation. Cells were infected with WH3 strain tachyzoites (MOI = 3) ± 100 μg/mL peptides for 6 h. Yellow puncta (RFP+GFP+ ) represent autophagosomes, while red puncta (RFP+GFP−) indicate autolysosomes. Scale bar: 10 μm. (**B and C**) Immunoblot analysis of autophagy-related proteins in RAW264.7 cells infected with WH3 (MOI = 3) ± peptide treatment (100 μg/mL, 2 h). (**C**) The expression levels of phosphorylated EGFR (Tyr1068) and AKT (Ser473), as well as LC3-II and LAMP1, were measured by Western blot. served as loading control. Blots are representative of three independent experiments. ns, not significant (*P* > 0.05). (**D and E**) Spatial analysis of LC3B recruitment. *P < 0.05, **P < 0.01, and ***P < 0.001 by Student’s *t*-test. (**D**) Confocal images showing peri parasitic accumulation of LC3B (red) around RH-GFP tachyzoites (green). The widefield panel (left) shows a zoomed-out view, with the boxed area indicating the region enlarged in the single-cell images (right). Scale bars: 10 μm (widefield panel); 10 μm (single-cell images). (**E**) Quantification of LC3B fluorescence intensity within 2 μm radius of 20 randomly selected parasites/condition. (**F and G**) Lysosomal trafficking assessment. (**F**) Confocal images showing periparasitic accumulation of LysoTracker staining (red) around RH-GFP tachyzoites (green). The widefield panel (left) shows a zoomed-out view, with the boxed area indicating the region enlarged in the single-cell images (right). Scale bars: 10 μm (widefield panel); 10 μm (single-cell images). (**G**) Quantitative analysis of lysosomal fluorescence intensity around individual parasites. All imaging data represent mean ± SEM of ≥ 3 experiments. ****P < 0.0001 by student’s *t*-test.

To elucidate the mechanism by which the C8 peptide counteracts *T. gondii* immune evasion mediated through the EGF domain of TgMIC6, we systematically analyzed autophagy modulation in host cells. PMA-differentiated THP-1 monocytes, pre-transduced with adenoviral mRFP-GFP-LC3 constructs, were infected with WH3 strains with or without CD154 co-stimulation, following pretreatment with C8 peptides. Quantitative analysis revealed significant enhancement of both autophagosome formation (GFP+/mRFP+ puncta) and autophagic flux (GFP− puncta indicative of lysosomal fusion) in C8-treated cells compared to WH3-infected controls (Fig. 4A). These findings were validated in RAW264.7 murine macrophages, where immunoblotting demonstrated upregulated expression of LC3-II and LAMP-1 in C8-treated, WH3-infected cells, concomitant with reduced phosphorylation of EGFR and AKT, signaling events characteristically induced by WH3 but attenuated by C8 (Fig. 4B and C). Confocal microscopy confirmed pronounced periparasitic accumulation of LC3B-positive vesicles in both THP-1 and RAW264.7 cells following C8 treatment (Fig. 4D and E), with parallel lysosomal recruitment (LysoTracker-positive vesicles) surrounding intracellular tachyzoites (Fig. 4F through I). These collective findings demonstrate that C8 peptide restores anti-*Toxoplasma* autophagy pathways potentially compromised by TgMIC6-mediated immune evasion, through coordinated modulation of both autophagosome biogenesis and lysosomal targeting.

## DISCUSSION

*T. gondii*, an opportunistic parasite, affects approximately 30% of the world’s population. Toxoplasmosis remains a significant global health challenge, with current therapeutic regimens exhibiting limited efficacy and notable side effects, underscoring the urgent need for novel treatment strategies. Contemporary drug discovery approaches, including structure-activity relationship optimization and high-throughput phage display screening, have emerged as powerful tools for identifying potential therapeutics. TgMIC6 represents a particularly promising target due to its dual role in mediating host cell invasion and subverting immune defenses through EGFR-dependent autophagy suppression (42, 44). Therefore, our study employed a rigorous phage display screening protocol against TgMIC6, which led to the identification of several candidate peptide inhibitors.

In this study, C8 exhibited significant therapeutic effects at concentrations of 100 μg/mL *in vitro* and 5 mg/kg *in vivo*. Notably, even at 300 μg/mL, C8 displayed no cytotoxicity toward Vero cells, and histopathological analysis revealed no organ lesions in WH3-infected BALB/c mice treated with 5 mg/kg C8 (Fig. S2). Furthermore, C8 dose-dependently inhibited *T. gondii* invasion and replication without inducing cytotoxicity. In addition, it markedly prolonged the survival of mice acutely infected with diverse parasite strains, demonstrating consistent cross-strain efficacy.

Importantly, C8 maintained efficacy against the WH3-Δ*mic6* mutant, suggesting target promiscuity that was subsequently confirmed by computational modeling. AlphaFold3 predictions identified high-affinity interactions not only with TgMIC6’s EGF domains but also with key invasion complex components including TgMIC2’s von Willebrand factor A (vWA) domain and its binding partner M2AP (46, 47). These structural insights explain the peptide’s ability to disrupt critical host-pathogen interactions. Therefore, we shifted our experimental focus from using Δ*mic6* as a simple control to validate specificity, toward using it as a tool to uncover complexity. Subsequently, it was determined that the peptide modulates intracellular signaling cascades, significantly reduces infection-induced phosphorylation of EGFR/AKT, and restores autophagic flux via upregulation of LC3/LAMP1.

A key finding of our study is the proposed interaction between peptide C8 and the TgMIC2/M2AP complex, as modeled by AlphaFold3. We acknowledge that AlphaFold3 has inherent limitations in predicting ligand-binding interactions with absolute certainty, and the absence of direct experimental validation, such as through surface plasmon resonance or the use of Δ*mic2* parasites, is a limitation of our current work. However, the tool we employed represents the current state-of-the-art in structure prediction. As demonstrated by its developers, AlphaFold3 achieves a significant leap in accuracy for predicting protein-ligand and protein-peptide complex structures compared to traditional methods (48). Furthermore, there is a strong precedent for utilizing the AlphaFold platform in preliminary studies of peptide-protein interactions. Models based on AlphaFold2, for example, have been widely adopted in peptide-based drug design and for generating mechanistic hypotheses (49, 50), demonstrating the considerable potential of such computational tools in guiding experimental research. Our work follows a similar strategy, employing the more advanced AlphaFold3 to generate a key, and highly credible, hypothesis regarding the interaction between the C8 peptide and the TgMIC2/M2AP complex. This model provides a valuable starting point and a robust framework for future studies to experimentally validate this interaction and refine the precise binding epitope. Naturally, subsequent studies will involve generating TgMIC2 and TgM2AP knockout strains for further experimental validation.

Another particularly intriguing and unexpected finding was the divergent effect of C8 treatment in the eyes. While the peptide significantly controlled infection in the brain, spleen, and heart, it was associated with a non-significant trend of increased parasite burden in the ocular tissue. We hypothesize that the unique immunoprivileged environment of the eye may underlie this phenomenon. It is possible that by potently suppressing the systemic infection and associated immunopathology, C8 treatment might have inadvertently modulated the immune response in a way that temporarily disrupted the delicate local immune equilibrium in the eye. Such a shift could potentially create a transient niche that is permissive for parasite persistence or even limited replication. This hypothesis is consistent with the concept of immune reconstitution, where effective systemic therapy can sometimes alter local immune dynamics with unintended consequences. Alternatively, differences in drug pharmacokinetics, such as inadequate penetration of the blood-ocular barrier or unique drug metabolism within the ocular tissue, could result in sub-therapeutic C8 concentrations, failing to exert direct anti-parasitic effects. These observations highlight the complexity of treating disseminated infections and underscore the critical need for future studies to specifically investigate the pharmacokinetics and local immunomodulatory effects of anti-*Toxoplasma* therapies within the ocular compartment.

The role of IFN-γ in toxoplasmosis is a double-edged sword. While essential for controlling parasite replication (51), an excessive and dysregulated IFN-γ response is a well-established driver of the cytokine storm responsible for lethal immunopathology during acute infection (52). In this study, the significant reduction in serum IFN-γ levels in C8-treated mice compared to succumbing controls (Fig. 2G) is a pivotal finding. We interpret this not as a failure to induce immunity, but as a successful attenuation of pathological hyperinflammation. The primary mechanism is likely twofold: first, by directly inhibiting parasite invasion and proliferation (Fig. 1 and 4), C8 drastically reduces the antigenic burden that triggers the overwhelming inflammatory cascade. Second, the consequent reduction in IFN-γ levels prevents the associated collateral tissue damage and disease sequelae. Thus, C8 treatment achieves a favorable immunological state by tilting the balance away from a lethal, hyperinflammatory response and toward a controlled, effective anti-parasitic immunity, as evidenced by the significantly improved survival despite lower overall IFN-γ. This highlights that the goal of therapy in acute toxoplasmosis is not merely to elevate immune activation, but to achieve pathogen control while avoiding immunopathology, a balance that C8 treatment successfully strikes.

The other key proposed mechanism of peptide C8 is the restoration of host autophagy to combat *Toxoplasma* infection. While our data demonstrate a compelling correlation between C8 treatment, the upregulation of LC3-II/LAMP1 enhanced lysosomal recruitment, and parasite clearance (Fig. S5), establishing a definitive causal relationship requires further investigation. A standard approach to validate such causality is through pharmacological inhibition of autophagy. However, in the context of our study, the use of common inhibitors like chloroquine presents a significant interpretative challenge. It is well-established that chloroquine possesses intrinsic, potent anti-*Toxoplasma* activity independent of its effect on host autophagy, as it directly damages the parasite’s membrane structures and inhibits its replication (53). Therefore, any experiment co-treating with C8 and chloroquine would be uninterpretable, as the resulting reduction in parasite burden could be attributed to chloroquine’s direct parasiticidal effect, the inhibition of autophagy, or a combination of both, thereby confounding the specific role of C8-induced autophagy. This constitutes a limitation of the present study. Future work to conclusively establish the functional contribution of autophagy should employ more specific genetic interventions, such as CRISPR/Cas9-mediated knockout of key autophagy genes (e.g., ATG5 or ATG7) in host cells. Such approaches would provide a cleaner system to dissect the necessity of the autophagy pathway for C8’s anti-parasitic efficacy without the complicating direct effects of pharmacological agents on the parasite itself.

Although our results suggest that the peptide C8 could serve as a promising anti-parasitic agent, some limitations remain. From a drug design perspective, the promiscuous targeting capability of C8 may represent an advantage in overcoming redundancy in parasitic invasion pathways. However, this same property necessitates careful assessment of potential off-target effects in human hosts. Additionally, we observed that C8 exhibits limited stability, losing efficacy in the acute infection model when stored beyond 6 months at −20°C or 3 months in solution at −80℃ (Fig. S4). Moreover, C8 showed no significant effect on chronic *T. gondii* infection, which may be attributed to structural constraints of the peptide or suboptimal administration methods. These findings underscore key challenges in the development of antiparasitic peptides, particularly regarding stability and therapeutic breadth.

Looking forward, future research should prioritize structural optimization of C8 to enhance its stability and pharmacokinetic profile. Strategies may include cyclization, incorporation of non-natural amino acids, or lipid conjugation to improve half-life and membrane permeability. Further mechanistic studies are also needed to fully elucidate the pathways through which C8 exerts its effects, particularly its impact on parasite replication and host immune signaling. *In vivo* studies using advanced delivery systems—such as nanoparticle carriers or sustained-release formulations—could help overcome the limitations observed in chronic infection settings. Ultimately, translational studies in larger animal models will be essential to evaluating the clinical potential of C8 or its derivatives.

In summary, this study not only provides a foundation for the development of novel anti-*T*. *gondii* therapeutics but also offers valuable insights for the design and application of peptide-based drugs.

### Conclusion

Through targeted phage display screening, we identified the C8 peptide as a potent, multi-target inhibitor of *T. gondii* host cell invasion. This compound exhibits an exceptional safety profile and demonstrates three complementary mechanisms of action: (i) direct blockade of micronemal protein function, (ii) restoration of compromised autophagic pathways, and (iii) modulation of pro-survival signaling cascades. While pharmacokinetic limitations currently restrict its clinical applicability against chronic infections, C8 represents a valuable lead compound for further development. This study significantly advances the therapeutic potential of peptide-based drugs against apicomplexan parasitic infections. Future research should focus on further optimizing the metabolic stability and tissue distribution properties of these peptides to establish a molecular design framework for next-generation anti-*Toxoplasma* agents.

## MATERIALS AND METHODS

### Preparation of peptides with affinity for TgMIC6

To develop specific TgMIC6-targeting peptides, recombinant TgMIC6 protein was first expressed and purified to serve as a target for phage display screening. The Ph.D. C7C Phage Display Peptide Library (New England Biolabs, USA) was employed, and three rounds of stringent biopanning consisting of affinity binding, washing, and competitive elution were performed to isolate high-affinity peptide binders (Fig. S1A). From the enriched phage clones, 10 distinct peptide sequences were identified. The corresponding linear peptides and their cyclized derivatives were synthesized via N- to C-terminal disulfide bond formation through cysteine-mediated oxidative cyclization (Shanghai Dechi Biosciences Co., Ltd., China), with all compounds purified to >95% homogeneity by reverse-phase HPLC and characterized by MALDI-TOF mass spectrometry. All 20 peptides were systematically designated for subsequent functional characterization (Fig. S1B).

### Parasites and cell culture

The RAW 264.7 macrophage cell line, Vero (Verda Reno) epithelial cells, and human foreskin fibroblasts (HFFs) were cultured in Dulbecco’s modified Eagle’s medium (DMEM, Gibco, USA), supplemented with 10% heat-inactivated fetal bovine serum (FBS, BioFroxx, Germany), 2 mM L-glutamine, and 50 μg/mL penicillin and streptomycin. THP-1 monocytes were cultured in RPMI 1640 medium (Gibco, USA), supplemented with 10% heat-inactivated fetal bovine serum, 2 mM L-glutamine, and 50 μg/mL penicillin and streptomycin. All cell lines were incubated at 37℃ in a humidified atmosphere containing 5% CO₂.

Tachyzoites of *T. gondii* were propagated in confluent HFF monolayers using DMEM with 3% (vol/vol) heat-inactivated FBS, 2 mM L-glutamine, and 50 μg/mL penicillin-streptomycin. Routine passaging of the parasites was performed by scraping and transferring them to fresh HFF monolayers. For experimental infections, tachyzoites were released from host cells by extrusion through a 27-gage needle. WH6 strain cysts were maintained *in vivo* by serial passage in Kunming mice.

### Invasion assay

The invasion efficiency of *T. gondii* strains was quantitatively assessed using an established two-color fluorescence assay (54). Briefly, HFF monolayers grown on coverslips in 24-well plates were inoculated with freshly egressed tachyzoites at a 3:1 parasite-to-host cell ratio and allowed to invade for 2 h, with experimental groups treated with 100 μg/mL peptide compound. Following three PBS washes to remove non-internalized parasites, cells were fixed with 4% paraformaldehyde (PFA) for 20 min and blocked with 3% bovine serum albumin (BSA) for 1 h. Extracellular parasites were specifically labeled through sequential incubation with rabbit anti-*T*. *gondii* glide-associated protein 45 (TgGAP45) polyclonal antibodies (1:1,000 dilution, kindly provided by Dr. Yonggen Jia, Beijing Tropical Medicine Research Institute, Beijing Friendship Hospital, Capital Medical University) and Alexa Fluor 594-conjugated anti-rabbit secondary antibodies (1:1,000, Thermo Fisher Scientific, USA) prior to membrane permeabilization. Subsequent to 0.2% Triton X-100 treatment for 20 min to permeabilize host membranes, total parasite populations (both intra- and extracellular) were stained using the same primary antibody followed by Alexa Fluor 488-conjugated secondary antibodies (1:1,000, Thermo Fisher Scientific, USA). Invasion efficiency was calculated as the ratio of intracellular parasites (Alexa Fluor 488-positive only) to total parasites per host nucleus, with quantitative analysis performed through immunofluorescence microscopy. This differential staining approach allowed precise discrimination between attached and internalized tachyzoite populations, with invasion rates expressed as the percentage of Alexa Fluor 488-positive parasites that lacked concurrent Alexa Fluor 594 staining relative to the total parasite count.

### Intracellular replication assay

HFF monolayers were cultured on coverslips in 24-well plates and infected with freshly egressed WH3 strain tachyzoites at a 1:1 parasite-to-host cell ratio for 36 h, with parallel treatment groups receiving 100 μg/mL peptide compound. Following infection, coverslips were fixed in 4% PFA for 20 min at room temperature and then stained using the Wright-Giemsa method for microscopic examination. Quantitative analysis was performed by enumerating tachyzoites within 100 randomly selected parasitophorous vacuoles (PVs) per experimental condition under oil immersion microscopy, with three independent replicates conducted to ensure statistical reliability. This standardized protocol allowed for precise assessment of parasite proliferation dynamics under compound treatment conditions while maintaining consistent host cell viability throughout the experimental timeframe.

### Double-labeled adenovirus detects autophagic flow

THP-1 cells were plated on glass coverslips in 24-well plates and differentiated into macrophage-like cells through 24-h stimulation with 100 ng/mL phorbol-12-myristate-13-acetate (PMA, MedChemExpress, China). Subsequently, the cells were transduced with adenoviral vectors encoding mRFP- and GFP-tagged LC3 proteins for 6 h to monitor autophagy flux, and then each group was stimulated with human CD154 (PeproTech, USA) for 12 h to activate immune signaling pathways. Freshly egressed tachyzoites were then introduced at a 3:1 parasite-to-cell ratio for 2-h invasion assays, with experimental groups treated with 100 μg/mL of the target peptide. Following fixation with 4% PFA for 20 min at room temperature, cellular nuclei were counterstained with 4′,6-diamidino-2-phenylindole (DAPI, Beyotime, China) for morphological reference. High-resolution images were captured at 63× magnification using a Zeiss LSM880 laser scanning confocal microscope equipped with appropriate filter sets for simultaneous detection of mRFP, GFP, and DAPI fluorescence signals. Quantitative image analysis was performed using ImageJ software with threshold-based segmentation and colocalization algorithms to determine autophagosome formation, with at least 20 cells analyzed per experimental condition across three independent replicates to ensure statistical significance.

### Cell viability

Vero cell monolayers were cultured in 96-well plates and exposed to serial concentrations of peptide C8 (1, 10, 100, 200, and 300 µg/mL) or pyrimethamine (1, 10, and 100 µg/mL) for 24 h to assess cytotoxicity. Following treatment, cell viability was quantified using the water-soluble tetrazolium salt assay, where 100 µL of 2-(2-methoxy-4-nitrophenyl)−3-(4-nitrophenyl)−5-(2,4-disulfophenyl)−2H-tetrazolium sodium salt (WST-8, GlpBio, China) was added to each well and incubated under light-protected conditions for 2 h at 37°C. The metabolic activity of viable cells was determined by measuring the absorbance at 450 nm using a microplate reader. Three independent experiments were performed with five technical replicates per condition to ensure statistical reliability, and the percentage of viable cells was calculated relative to untreated controls after subtracting blank well readings.

### Immunofluorescence

To assess LC3B expression, THP-1 and RAW264.7 cells were plated on glass coverslips in 24-well plates, with THP-1 cells first differentiated via 24-h exposure to 100 ng/mL PMA followed by 12-h stimulation with human CD154, while RAW264.7 cells received 12-h mouse CD154 (PeproTech, USA) treatment. Subsequently, freshly egressed RH-GFP strain tachyzoites were introduced at a 3:1 parasite-to-cell ratio for 2-h invasion assays, with parallel treatment groups receiving 100 μg/mL C8 peptide. Following three PBS washes to remove extracellular parasites, cells were fixed with 4% PFA for 20 min, permeabilized with 0.1% Triton X-100 for 15 min at room temperature, and blocked with 3% BSA for 1 h at 37°C. Immunofluorescence staining was performed using rabbit anti-LC3B primary antibodies (1:200, Sigma, USA) overnight at 4°C, followed by Alexa Fluor 594-conjugated anti-rabbit secondary antibodies (1:1,000, Thermo Fisher Scientific, USA) for 1 h at 37°C, with nuclear counterstaining using DAPI. For lysosomal detection, identically treated cells cultured in confocal dishes (Beyotime, China) were stained with Lyso-Tracker Red (1:15,000, Beyotime, China) for 60 min at 37°C under 5% CO₂, followed by Hoechst 33,342 (1:100, Beyotime, China) for 5 min to visualize nuclei. All samples were imaged at 40× magnification using a Laser Confocal Microscope (LSM880, Zeiss, Germany), with quantitative analysis of fluorescence intensity and puncta formation performed using ImageJ software with standardized thresholding protocols across three biological replicates.

### Western blotting

RAW264.7 cells were processed using the same methods as those described for LC3 and lysosome detection. Cells were collected by centrifugation at 4°C and washed with cold PBS, followed by lysis using RIPA lysis buffer containing 1mM EDTA, phosphatase inhibitors, and protease inhibitors. After 30 min of lysis on ice, the cell lysates were centrifuged at 12,000 × *g* for 30 min at 4°C, and the supernatant was collected after centrifugation. The supernatant was mixed with 5× loading buffer at a ratio of 4:1 and boiled at 100°C for 10 min. Proteins were separated using SDS-PAGE, transferred to a polyvinylidene fluoride (PVDF, Merck, Germany) membrane, and then blocked in Tris buffer containing 3% BSA and 1% Tween 20. Primary antibodies against LC3B (1:1,000), pEGFR (1:1,000), pAKT (1:1,000), LAMP-1 (1:1,000) (all from Cell Signaling Technology, USA), and β-actin (1:8,000, biosharp, China) were incubated overnight at 4°C. Subsequently, the corresponding secondary antibodies (1:10,000, SparkJade, China) were incubated at 37°C for 1 h. After washing with TBST, signals were detected using an ECL kit (Sparkjade, China) and visualized on a ChemiDoc XRS+ (Bio-Rad, USA).

### Animal experimentation

Six to eight weeks old female BALB/c mice were obtained from the animal center of Anhui Medical University and housed under specific pathogen-free conditions with *ad libitum* access to autoclaved water and standard rodent chow. Following a 1-week acclimatization period, the mice were randomly allocated into three experimental groups (*n* = 6 per group), a PBS-treated control group, a pyrimethamine (PYR), and a peptide-treated intervention group, to systematically evaluate the therapeutic efficacy of the candidate peptide against *T. gondii* infection. Tachyzoites from distinct parasite strains including WH3 (10^3^ parasites/mouse), WH3-Δ*mic6* (10^3^ parasites/mouse), RH (10^1^, 10^2^, and 10^3^ parasites/mouse), and ME49 (10^6^ parasites/mouse) were administered via intraperitoneal injection to establish infection. Concurrent with parasite inoculation, mice in the treatment group received an initial intraperitoneal bolus of 5 mg/kg peptide solution, followed by daily tail vein injections of either sterile PBS or 5 mg/kg peptide solution for four consecutive days. All animals were monitored twice daily for clinical signs of toxoplasmosis (including piloerection, lethargy, and neurological symptoms), with survival times. Humane endpoints were strictly enforced according to institutional animal welfare guidelines, with moribund animals euthanized by CO_2_ asphyxiation followed by cervical dislocation.

To quantify *T. gondii* burden in multiple organs at distinct time points post-infection, cardiac, cerebral, ocular, and splenic tissues were aseptically collected from both control and peptide-treated mice on days 3, 5, and 7 following intraperitoneal inoculation with 10³ WH3 strain tachyzoites. Genomic DNA was isolated from homogenized tissues using a commercial DNA extraction kit (Accurate Biology, China) according to the manufacturer’s protocol. Parasite load was assessed by SYBR Green I-based quantitative real-time PCR (qPCR) using the following primer sets: *T. gondii* B1 gene (forward: 5′-TGCATAGGTTGCAGTCACTG-3′; reverse: 5′-TCTTTAAAGCGTTCGTGGTC-3′) and mouse GAPDH as an endogenous control (forward: 5′-CATGGCCTTCCGTGTTCCTACC-3′; reverse: 5′-CCTGCTTCACCACCTTCTTGAT-3′). Reactions were performed in 20 μL volumes containing 10 μL of 2× SYBR Premix Ex Taq (TargetMol, USA), 0.4 μM of each primer, and 2 μL of template DNA on a QuantStudio 6 Flex system (Applied Biosystems) with the following cycling parameters: 95℃ for 30 s, followed by 40 cycles of 95℃ for 10 s and 60℃ for 30 s, with a final melt curve analysis stage (95℃ for 10 s, 65℃ for 1 min, then gradual increase to 97℃ at 0.2℃/s). Specificity of amplification was confirmed by melt curve analysis and agarose gel electrophoresis of representative products. Cycle threshold (Ct) values were analyzed by comparative ΔΔCt method, with parasite DNA levels normalized to GAPDH and expressed as fold-changes relative to baseline controls, with all reactions performed in technical triplicates.

To evaluate the therapeutic efficacy of the candidate peptide against chronic *T. gondii* infection, three experimental groups of mice (*n* = 6–8 per group) were orally inoculated with 20 WH6 strain tissue cysts suspended in 200 μL of PBS using a ball-tipped gavage needle. Beginning 24 h post-infection, each group received daily interventions for five consecutive days: Group 1 received 50 mg/kg pyrimethamine (PYR, Sigma-Aldrich, USA) via oral gavage as positive control; Group 2 received 200 μL sterile PBS via tail vein injection as negative control; and Group 3 received 5 mg/kg peptide solution in equivalent volume via tail vein injection as experimental treatment. Clinical monitoring was performed twice daily with assessment of survival rates. At the experimental endpoint (30 days post-infection), surviving mice were euthanized by CO_2_ asphyxiation followed by cervical dislocation, and whole brains were aseptically collected. Brain tissues were homogenized in 1 mL PBS, and 10 μL aliquots of homogenate were mounted on glass slides under 18 × 18 mm coverslips. Cyst quantification was performed by blinded investigators using phase-contrast microscopy (Zeiss, Germany) at 200× magnification, with counts normalized to total brain tissue.

### Enzyme-linked immunosorbent assay

The concentration of IFN-γ in the sera of each mouse infected with the parasite was measured using a mouse ELISA Kit (Boster, China) according to the manufacturer’s instructions. Optical density values were detected at 450 nm, and the concentration of each sample was calculated using the curve measured by the standard sample.

### Molecular docking

Molecular docking simulations were conducted to explore the potential interactions between the target protein and ligand molecules. The sequence of the target protein was obtained from ToxoDB (http://toxodb.org/toxo/app). Molecular docking was performed using AlphaFold 3 (https://alphafoldserver.com), and the results were visualized using PyMOL 3.1.

## SUPPLEMENTAL MATERIAL

Supplemental figures spectrum.02218-25-s0001.pdf Fig. S1 to S5.

ASM does not own the copyrights to Supplemental Material that may be linked to, or accessed through, an article. The authors have granted ASM a non-exclusive, world-wide license to publish the Supplemental Material files. Please contact the corresponding author directly for reuse.
