# Supplementary material for: A novel anti-Toxoplasma peptide suppresses parasite invasion and rescues host autophagic defenses
Source: Microbiol Spectr. 2025 Dec 29;14(2):e02218-25. doi: 10.1128/spectrum.02218-25 (PMC12889027; doi:10.1128/spectrum.02218-25)
Supplement: Supplemental figures — Fig. S1 to S5. [file spectrum.02218-25-s0001.pdf]

A

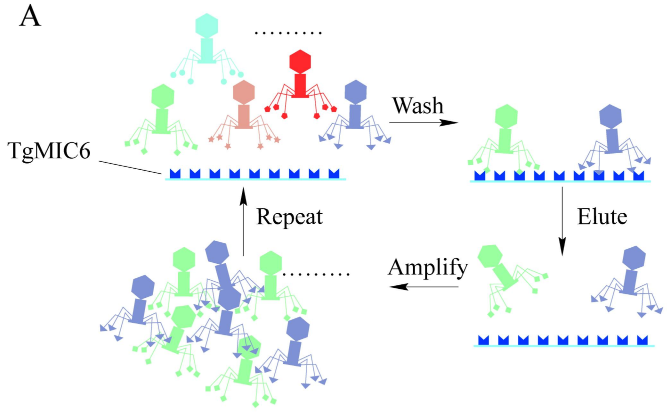

B

Number	Serial number	Sequence
A1	P101797(CC-9-10)	CKHGLDTRC
A2	P101796(CC-9-10(O))	CKHGLDTR-Cys
A3	P101788(CC-9-6(O))	CSKAPHAV-Cys
A4	P101789(CC-9-6)	CSKAPHAVC
A5	P101784(CC-9-4(O))	CFNTPDKR-Cys
A6	P101785(CC-9-4)	CFNTPDKRC
A7	P101790(CC-9-7(O))	CNVAGLQF-Cys
A8	P101791(CC-9-7)	CNVAGLQFC
A9	P101786(CC-9-5(O))	CYATSYPH-Cys
A10	P101787（CC-9-5)	CYATSYPHC
C1	P101783(CC-9-3)	CMTNAPSNC
C2	P101783(CC-9-3(O))	CMTNAPSN-Cys
C3	P101778（CC-9-1(O))	CHTTPKKL-Cys
C4	P101779(CC-9-1)	CHTTPKKLC
C5	P101794(CC-9-9(O))	CSLYLTPN-Cys
C6	P101795(CC-9-9)	CSLYLTPNC
C7	P101792(CC-9-8(O))	CYAGDRPG-Cys
C8	P101793(CC-9-8)	CYAGDRPQC
C9	CC-9-2(O)	CVHDWLSP-Cys
C10	CC-9-2	CVHDWLSPC

Supplementary Fig. 1 Preparation of peptides with affinity for TgMIC6. (A) Schematic representation of the phage display biopanning strategy. TgMIC6 protein was exogenously expressed and used as the target protein in phage library display technology. Multiple rounds of phage selection were performed using affinity washing methods. The above procedures were repeated after expanding cultivation through *Escherichia coli* to further screen and obtain peptide fragments that interact with the target protein. (B) Tabulated sequences of the 10 lead peptide candidates identified through next-generation sequencing of enriched phage pools. Peptides are designated as A1-A10 and C1-C10, and cyclized forms generated through formation of disulfide bridges between N- and C-terminal cysteine residues. All sequences were verified by Sanger sequencing and synthesized to > 95% purity.

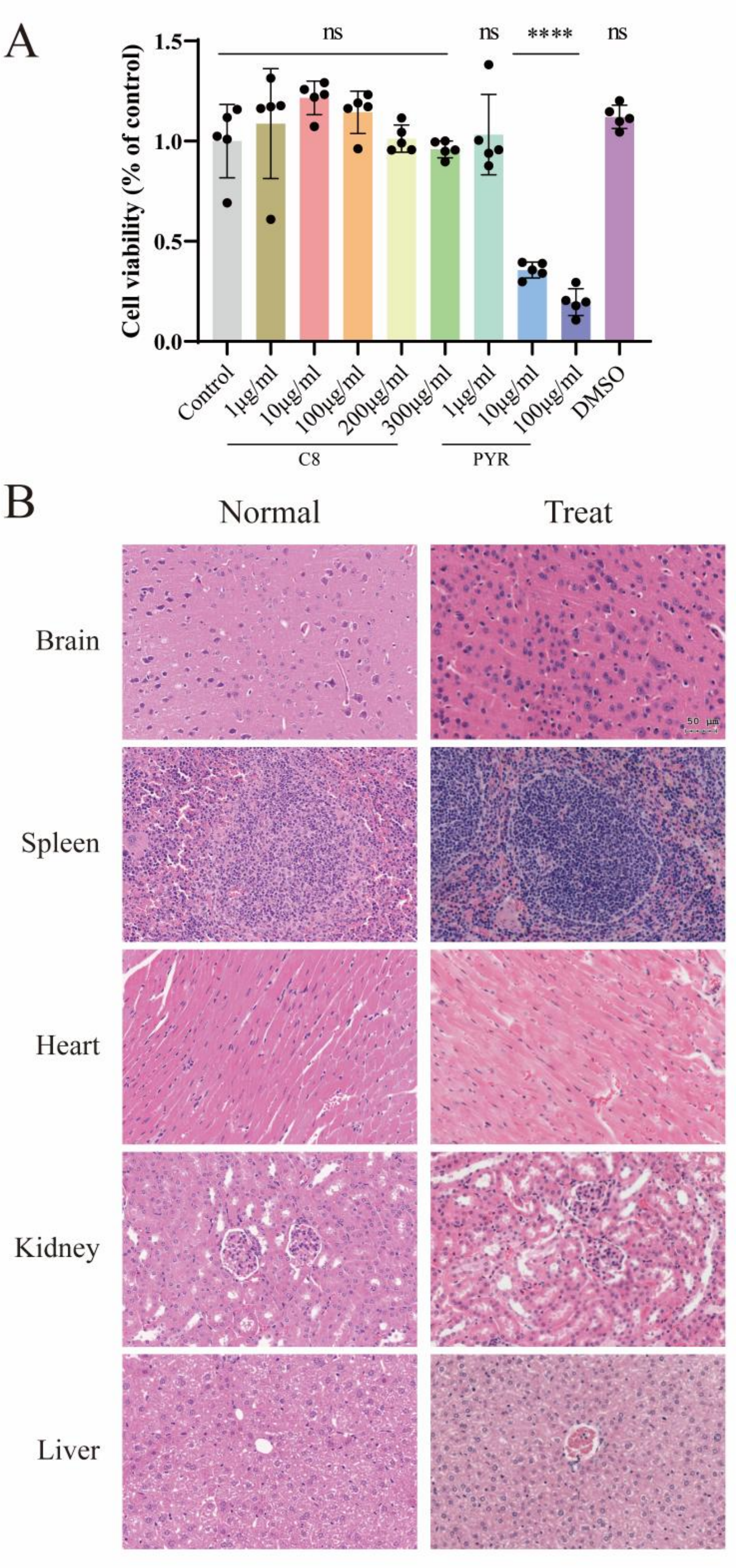
A
Cell viability (% of control)
ns
ns
****
ns
Control
1μg/ml
10μg/ml
100μg/ml
200μg/ml
300μg/ml
1μg/ml
10μg/ml
100μg/ml
DMSO
C8
PYR
B
Normal
Treat
Brain
Spleen
Heart
Kidney
Liver

11

Supplementary Fig. 2 Safety profile assessment of C8 peptide *in vitro* and *in vivo*. (A) Cytotoxicity evaluation in Vero cells exposed to peptide candidates (1-300 μg/mL) or pyrimethamine (1-100 μg/mL) for 24 h. Cell viability was quantified by CCK-8 assay (absorbance at 450 nm), with values normalized to untreated controls (100% viability). Data represent mean ± SD of triplicate experiments. (B) Histopathological analysis of major organs (brain, spleen, heart, kidney, liver) from WH3-infected BALB/c mice following 5-day C8 peptide treatment (5 mg/kg/day). Tissues were sectioned and stained with H&E, showing preserved tissue architecture without evidence of drug-induced pathology. Images are representative of n=6 mice/group. Scale bar: 10 μm.

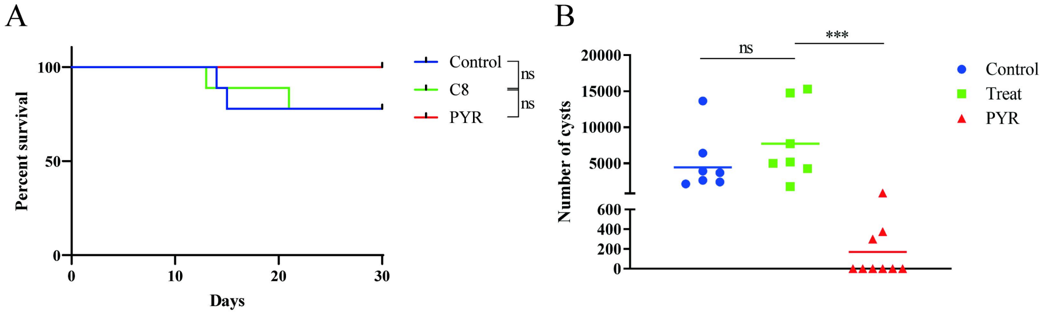

Supplementary Fig. 3 Therapeutic evaluation of C8 peptide in chronic toxoplasmosis. (A) Kaplan-Meier survival curve of Kunming mice (n=8/group) orally inoculated with 20 WH6 tissue cysts and treated with C8 peptide (5 mg/kg/day, i.v. for 5 days) or PBS control. Monitoring continued for 30 days post-infection, with survival rates assessed twice daily. Statistical analysis showed no significant difference between groups (p >0.05 by Gehan-Breslow-Wilcoxon test). (B) Quantitative assessment of brain cyst burden at 30 days post-infection. Cysts were enumerated in 20 μL homogenates from whole brain tissue under light microscopy (400× magnification). Data represent mean ± SEM (n=7 brains/group). No significant

reduction was observed in C8-treated versus control mice (p = 0.1999 by unpaired t-test).

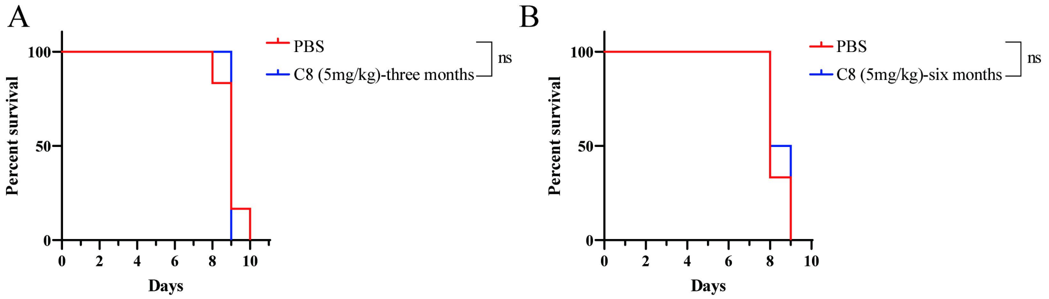

Supplementary Fig. 4 Stability of C8 peptide. (A-B) Kaplan-Meier survival analysis. (A) Treatment with C8 peptide solution stored at -80°C for 3 months for 10^3 WH3 infections in BALB/c mice. (B) Treatment with C8 peptide stored at -20°C for 6 months for 10^3 WH3 infections in BALB/c mice.

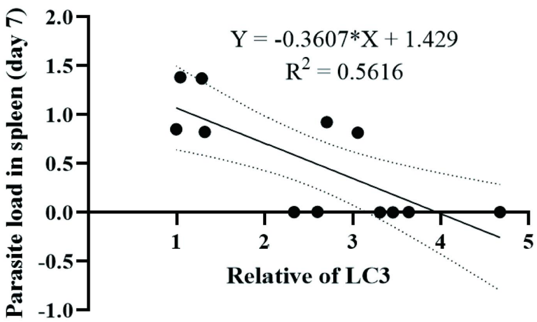

Supplementary Fig. 5 Correlation analysis between autophagy induction and anti-parasitic efficacy. Correlation between splenic parasite load on day 7 post-infection and relative LC3 expression. The X-axis represents the relative level of intracellular LC3 expression, and the Y-axis represents the splenic parasite load on day 7 post-infection. R^2 = 0.5616.
